# A randomized controlled trial of a coping-focused family resilience intervention program for breast cancer patients: Study protocol

**DOI:** 10.3389/fpsyg.2022.968154

**Published:** 2022-10-14

**Authors:** Jie Gao, Jia-Xin Li, Wei-Ying Chen, Jiang-Yan Song, Meng-Ke Zhou, Shan-Shan Zhang, Hui-Ping Li

**Affiliations:** ^1^School of Nursing, Anhui Medical University, Hefei, Anhui, China; ^2^Department of Nursing, Anhui Provincial Hospital, Hefei, Anhui, China; ^3^Department of Nursing, Clinical College of Anhui Medical University, Hefei, Anhui, China

**Keywords:** breast cancer, family resilience, intervention, protocol, randomized control trail

## Abstract

**Background:**

Breast cancer patients and their families are under various pressures in the process of disease diagnosis and treatment, which seriously threaten their physical and mental health. Findings from existing research suggest that good family resilience can help breast cancer families better adapt and cope with adversity and challenges. However, there are only a few intervention studies on family resilience and no intervention studies on resilience among the families of breast cancer patients. Therefore, this study aims to explore appropriate measures to improve the resilience level of breast cancer families and help them better cope with the disease.

**Objective:**

The purpose of this study protocol is to demonstrate a coping-focused family resilience intervention to increase the level of resilience in the families of breast cancer patients and help them better cope with adversity.

**Methods:**

The trial will recruit 80 breast cancer families and randomly assign them to experimental and control groups in a 1:1 ratio. The control group will receive routine care, and the intervention group will receive a 6-week one-on-one online family resilience intervention based on the control group. Two groups of subjects will be assessed at baseline and at 1 week, 1 month, and 3 months after the intervention. The primary outcome is family resilience, and the secondary outcomes are coping style, social support, family disease burden, and levels of anxiety and depression.

**Expected results:**

We hypothesized that after the intervention, the intervention group would have significantly higher levels of family resilience than the preintervention and control groups. In the intervention group, other aspects related to family resilience, such as family disease burden and anxiety and depression levels of patients and their families, were significantly alleviated, and disease coping and social support levels were improved accordingly.

**Discussion:**

If the program works, it can help breast cancer families identify family strengths and resources to proactively address challenges so that families can successfully navigate the crisis and patient and family recovery can be facilitated. It can also provide a practical path for clinical workers to help breast cancer families adjust rationally.

**Clinical Trial Registration:**

This study has been registered in the Chinese Clinical Trials Registry (Registration Number: http://www.chictr.org.cn/ChiCTR2100052108).

## Introduction

According to Global Cancer Statistics (GLOBOCAN), there were approximately 2.3 million new cases of breast cancer worldwide in 2020, ranking the disease first among all types of cancer (Sung et al., 2021). Global Cancer Survival Data (CONCORD-3) shows a continued increase in the 5-year net survival rate for breast cancer (Allemani et al., 2018). The 5-year net survival rate of Chinese breast cancer patients is up to 84% (Zeng et al., 2018), and the age of onset tends to be younger (Chen et al., 2018). With the increasing incidence of breast cancer, the improvement of survival rates, and the younger age of onset, people are paying increasing attention to the quality of life of breast cancer patients.

The diagnosis and treatment of breast cancer not only causes great distress to the patient physically, psychologically and socially but induces many physical, psychological and social problems for family members (Wagner et al., 2006; Rabin et al., 2009; Yu and Sherman, 2015; Feeney et al., 2018; Tsaras et al., 2018; Boamah et al., 2021). A series of problems faced by patients and their families often lead to unbalanced family relationships and reduced family intimacy and function that challenges patients and their families (Chen, 2015). It easily causes a family crisis and affects the patient’s recovery and quality of life. In China, the incidence of breast cancer has been increasing annually in recent years, and the incidence of breast cancer combined with depression has shown a significant upward trend (Su, 2021; Sung et al., 2021). The diagnosis and treatment of breast cancer has placed a heavy burden on family members, and the marital quality of breast cancer patients has also been seriously damaged (Pan et al., 2022; Wu et al., 2022).

Meanwhile, when faced with the severe challenges of breast cancer, some families are able to adapt effectively and make positive changes that allow them to recover and grow (Markman et al., 2020). Research shows that family resilience plays an important role. Family resilience is an important protective factor for individual resilience (Zhang, 2015), which can directly or indirectly improve individual resilience, patients’ quality of life and disease coping ability, and patients’ subjective well-being and mental health (Chen et al., 2019). During the formation and development of family resilience, breast cancer families have experienced positive changes in disease cognition, member psychology, family atmosphere, and family relationships (Wu, 2020). However, research on breast cancer family resilience has mostly focused on the relationship between related factors, and no further interventions to improve family resilience have been reported. Therefore, it is important to design effective interventions to increase the level of resilience among the families of breast cancer patients in China.

Family resilience was first proposed by Walsh based on the concept of psychological resilience, which elevates resilience from the individual level to the family level (Walsh, 2003). Dai Yan believes that family resilience is not only a trait and ability to help families adjust and adapt under pressure and adversity but also a process of helping the entire family dynamically adjust to better adapt and cope with adversity and changes (Dai, 2008).

At present, family resilience has attracted increasing attention from scholars worldwide, and intervention research on family resilience is still in its infancy. From a previous literature review, only nine family resilience intervention studies have entered clinical trials (Weine et al., 2003; Chartier et al., 2010; Lim and Han, 2013; Inci and Temel, 2016; Miran, 2016; Dorell et al., 2017; Wang, 2017; Faarup et al., 2019; Yu et al., 2020), of which only three are randomized controlled studies (Inci and Temel, 2016; Wang, 2017; Yu et al., 2020), and there is no report on family resilience intervention studies for breast cancer patients. Most family resilience intervention studies are in the construction or preexperimental stage of the intervention plan (Riley et al., 2008; Foster et al., 2012; Lim and Han, 2013; Miran, 2016; Zhao, 2016; Dorell et al., 2017). The construction process and content of the intervention programs have not been reported in detail. Current research on family resilience interventions often uses multifamily group meetings involving patients and family members from multiple families (Weine et al., 2003; Lim and Han, 2013; Inci and Temel, 2016). The meetings mainly focus on disease knowledge, stress and emotion management, coping with family problems, and family communication methods. Some studies focus on family communication interventions (Dorell et al., 2017; Faarup et al., 2019). Although the findings suggest that most interventions had some effect on improving the study subjects’ family resilience levels and reducing related psychosocial problems, the format of multifamily group meetings may not be effective in China.

Due to the influence of traditional Chinese culture and the introverted nature of Chinese people, people are not used to expressing their feelings and sharing family problems publicly. This is consistent with what we learned earlier. Patients and their families prefer one-on-one family meetings. Therefore, one-on-one family meetings may be more effective than multifamily group meetings. Meanwhile, the postoperative treatment and follow-up of patients are mostly performed in local hospitals or outpatient clinics, and there is no time to participate in an intervention. In addition, repeatedly making appointments for patients and their families to come to the hospital to participate in an intervention increases the burden on the family and the risk of contracting COVID-19. Therefore, online meetings can solve these problems very well. The online meeting time and location are more flexible. Participants only need to find a relatively quiet place to join the meeting. The meeting time can also be adjusted if participants require it. Additionally, online meetings can be recorded. If some participants are absent due to personal reasons, they can learn about the content of the meeting from the video. Online meetings greatly improved the implementability and convenience of intervention programs.

Among the current family resilience intervention research, some studies are based on family resilience theory (Lim and Han, 2013; Inci and Temel, 2016; Miran, 2016), and some studies are based on other related theories (Chartier et al., 2010; Dorell et al., 2017; Wang, 2017; Faarup et al., 2019; Yu et al., 2020). Different theoretical foundations have different intervention programs, and the intervention effects also have certain differences. To this end, our research group Wu Dy used grounded theory to study the formation process of family resilience of breast cancer patients, modeled the family resilience process of resilience among the families of breast cancer patients, and laid a theoretical foundation for formulating a breast cancer family resilience intervention program (Wu, 2020).

Wu Dy′s family resilience process model of breast cancer patients describes the attitudes, strategies and influencing factors of Chinese families coping with a family member’s breast cancer (Wu, 2020). The process model shows that the formation of family resilience is affected by multiple protective factors, of which the coping process is the core category of the family resilience process model. The coping process dynamically modulates changes in disease perception and family resilience outcomes during the family resilience process and plays a leading role in the formation and development of family resilience. The coping process is also a game process of various facilitating and hindering factors, helping families to better adjust, adapt and cope with adversity and changes. Therefore, the coping process becomes the core and focus of family resilience interventions.

Walsh’s family resilience framework places the family at the core of a unit, emphasizing that family resilience is the result of the interaction of individuals, families and societies (Walsh, 2003). The theory divides the core of family resilience into three key processes: belief systems, organizational patterns, and communication/problem-solving processes. Many key elements of these three processes, such as meaning making, flexibility, and open emotional expression, can be intervened. The model has a relatively simple structure and is easy to operate, which is more suitable for clinicians assessing and intervening in patients’ family conditions, and it provides a more concise and easy-to-operate strategy and framework for family resilience intervention. Therefore, we should focus on the coping process in Wu Dy′s family resilience process model of breast cancer patients and use Walsh’s family resilience theory as a framework to construct a family resilience intervention plan for families of breast cancer patients in the Chinese context.

This intervention plan was based on relevant theories and combined with literature research to form the first draft of the family resilience intervention plan for Chinese breast cancer patients. Qualitative interviews were then conducted with breast cancer patients and their families, doctors and nurses in relevant departments. According to their comments, the content of the intervention plan was adjusted, and a revised draft of the intervention plan was formed. Experts in relevant fields were then contacted *via* email for two rounds of expert consultation on the content of the intervention program. According to the opinions of experts, the content of the intervention plan was adjusted for the second time, and the final draft of the family resilience intervention plan for breast cancer patients was formed. Finally, the intervention program is put into clinical practice for large-scale randomized controlled trials.

This study aimed at the family crisis of breast cancer patients and based on rigorous evidence, a coping-focused family resilience intervention program (CFFRI) for Chinese breast cancer patients will be validated. If the intervention proves effective, it could be used to increase the family resilience of breast cancer patients and help them better cope with crises and challenges.

### Hypotheses

The research hypotheses are as follows:

- After the intervention, the family resilience level of the intervention group will be significantly improved compared with that before the intervention.
- After the intervention, the family resilience level of the intervention group will be significantly improved compared with that of the control group.
- After the intervention, other aspects related to family resilience in the intervention group, such as the family disease burden and anxiety and depression levels of patients and their families, will be significantly relieved, and the level of disease coping and social support will be improved accordingly.

## Materials and methods

### Study design

**Figure 1:**
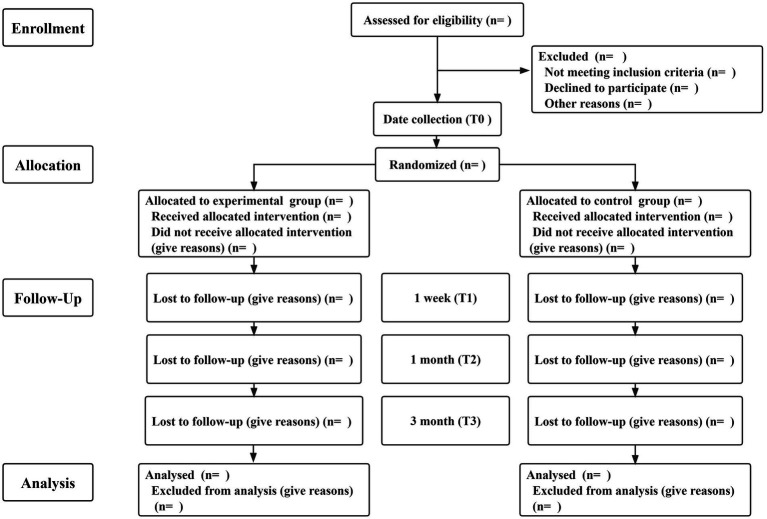
Consolidated Standards of Reporting Trials (CONSORT) flow diagram.

Due to the nature of the experiment, the interventionists could not be blinded in this study, so this study used a single-blind randomized controlled trial. Subjects will be included in the study in strict accordance with the inclusion and exclusion criteria. Eligible patients and their families will be included in the study according to their wishes. Participating families will be randomly divided into a control group and an experimental group according to computer-generated random numbers, with a distribution ratio of 1:1 (Figure 1). The control group will receive usual care. The experimental group will receive a family resilience intervention on the basis of usual care that will blind participants, recruiters and data collectors. This study constructs a family resilience intervention plan for breast cancer patients in strict accordance with the SPIRIT 2013 and CONSORT 2010 statement (Chan et al., 2013; Eldridge et al., 2016).

### Setting and recruitment

Subjects will be recruited from relevant departments of three general hospitals in Anhui Province and offered gifts and free medical exams. Recruiters will present the study to breast cancer families who meet the study criteria. If patients and their families are interested in participating in this study, they will be asked to sign informed consent forms and randomly assigned. Investigators should explain to participating families that the study will not adversely affect them and that participating families can opt out of the study at any time.

### Participants

#### Inclusion criteria for patients

- Women with first pathological diagnosis of breast cancer
- 18 years old and older
- Undergoing surgery
- Using a smartphone
- Good understanding and communication skills, no history of mental illness
- Informed consent, willing to cooperate

#### Exclusion criteria for patients

- Having other serious medical conditions
- Participation in other psychological interventions

#### Inclusion criteria for family members

- A fixed patient caregiver (first-degree relatives)
- 18 years old and older
- Good understanding and communication skills, no history of mental illness
- Using a smartphone
- Informed consent, willing to cooperate

#### Exclusion criteria for family members

- Having serious medical conditions
- Participation in other psychological interventions

### Sample size

Study sample size calculated by PASS15. Based on previous survey results (Lim and Han, 2013), family resilience measured by the Family Resilience Index (FHI) changed by 0.29 points before and after the intervention with a statistical power of 0.9, 2.5% significance, and using a one-tailed test to calculate a sample size of 36 families in each group (72 families in total; McCubbin et al., 1996). Assuming a 10% dropout rate in the intervention, each group would need to include 40 families (80 families in total). The sample size calculation formula is as follows:


$$n=\frac{{\left({Ζ}_{\alpha }+{Ζ}_{\beta }\right)}^{2*}2\sigma^{2}}{\delta^{2}}$$

A series of measures will be taken to reduce attrition during the intervention. First, during the intervention recruitment phase, the patient’s attending physician or other medical staff will be invited to answer the patient’s and family’s concerns to increase participants’ trust in the program. Second, a WeChat group will be established where researchers answer questions and share disease-related knowledge with participants. The third is to invite breast cancer treatment experts and nursing experts to control the quality of the entire intervention process, and timely answer questions from patients and their families about disease treatment and care. Fourth, during the intervention, participants can get in touch with the research team to get answers to any difficulties involved in the implementation and application of the intervention content. Finally, at the end of the program, the research team will present the participants with a nice gift to express their gratitude.

### Randomization

Eighty random numbers will be generated by a computer and randomly divided into an intervention group and a control group with a distribution ratio of 1:1. The assigned numbers will be sealed in 80 opaque envelopes. Recruiters will enroll research subjects who meet the research conditions, agree to participate in the research, and sign an informed consent form. Subjects will randomly draw envelopes, enter their assigned groups, and have baseline measurements taken. Participant recruitment and assignment will be done by two independent researchers, blinding participants, recruiters and data collectors.

### Intervention

#### Control group

Families in the control group will receive usual care. Usual care includes the following: First, the issuance of health handbooks, which include breast cancer-related knowledge, postoperative rehabilitation exercise guidance, clinical nursing practice guidelines for preventive behavior of breast cancer-related lymphedema and a breast cancer postoperative follow-up checklist. Second, a WeChat group will be established where researchers answer questions and share disease-related knowledge with participants. Finally, Everyone will be encouraged to share positive experiences related to the disease in the WeChat group to encourage and support each other.

#### Experimental group

The experimental group will implement one-on-one online family resilience intervention on the basis of usual care. Intervention implementation will be led by a professionally trained nursing graduate student and a breast cancer-related unit nurse. A breast cancer therapist and a psychobehavioral specialist will guide and supervise the implementation of the intervention. The entire intervention process includes a preintervention program introduction, six family meetings, and a postintervention summary. The frequency of interventions is weekly, with each meeting lasting 25–30 min. The first meeting, combined with patient follow-up time, will be an face-to-face family meeting with participating families, and the next five meetings will be online family meetings. The first follow-up visit to the hospital after surgery is usually approximately 1 week after surgery. A quiet, undisturbed environment will be chosen for family meetings. The specific time for each family meeting must be agreed upon in advance with participating families.

Before the intervention begins, the researcher will brief the intervention group families about the program and build a relationship of trust with them. Assessors will collect baseline data, and another researcher will teach participants to use online video software. The first family meeting will be held 1 week after the patient’s surgery, and the theme will be understanding breast cancer and family resilience. The second family meeting will be held 2 weeks after surgery, and the theme will be remaining positive. The third family meeting will be held 3 weeks after surgery, and the theme will be discovering available resources. The fourth family meeting will be held 4 weeks after surgery, and the theme will be improving family cohesion. The fifth family meeting will be held 5 weeks after surgery, and the theme will be facilitating effective communication. The sixth family meetings will be held 6 weeks after surgery, and the theme will be family problems and coping. The themes of the six meetings stemmed from Walsh’s family resilience framework. The themes for the first and second meetings were from the belief systems, the themes for the third and fourth meetings were from the organizational patterns, and the themes for the fifth and sixth meetings were from the communication/problem-solving process. Formation of the main contents of the six-week intervention plan through a literature review. Then, the form and content of the intervention plan were adjusted through structured interviews with patients, family members, and medical staff in breast cancer-related departments. Finally, through two rounds of expert consultation, the content of the intervention plan was revised to ensure the scientificity and feasibility of the intervention plan.

One day before each meeting, meeting materials will be sent to the participants of the intervention group to inform them of the content of the meeting in advance. Two to 3 min before each meeting, there will be a review to summarize the content of the previous meeting. During the meeting, families of the intervention group will be guided to actively participate in the discussion through videos, pictures, questions, etc., to increase participation. After the meeting, participating families will be encouraged to review the content of the meeting through homework to improve the effect of the intervention. One week after the end of the intervention, participating families will be invited to assess the entire intervention process, and the assessors will collect postintervention data and give gifts to families to show appreciation for their participation. Details of the intervention plan are shown in Table 1.

**Table 1:** Outline of the coping-focused family resilience intervention program (CFFRI).

Unbreakable Belief - Know yourself and the enemy (First week after surgery)	
Theme	Understand Breast Cancer and Family Resilience
Form	Face-to-face family meeting
Target	Learn about breast cancer and how it affects families. Understand the concept and meaning of family resilience.
Content	Ask the patient and primary caregiver to share some of their questions and concerns after patients becomes ill. Explain breast cancer related knowledge to patients and their families in the form of pictures, text and videos. Combine video materials and family discussions to help family members understand the characteristics of breast cancer families and help each family member understand the changes in family roles. Explain the definition and importance of family resilience to patients and their families in a graphic and textual manner.
Homework	Review the content of this meeting through electronic meeting materials. Patients and primary caregivers share with researchers their views on breast cancer and the main effects of the disease on themselves or their families (*via* voice or text message or phone call).
Unbreakable Belief - Face with smile (Second week after surgery)	
Theme	Remain Positive
Form	Online family meeting
Target	Breast cancer treatment, life and psychology and other aspects care and precautions. Master the basic methods to relieve stress and soothe emotions.
Content	A brief recap of last week’s meeting. Let the primary caregiver to review issues encountered during the patient’s care over the past week. Combined with pictures and texts, it provides nursing methods and precautions for different treatments for breast cancer patients and their families. Have patients and primary caregivers share their emotions and stress over the past week. Help patients and primary caregivers learn ways to manage stress and emotions to stay positive and optimistic by combining pictures, text and videos.
Homework	Review the content of this meeting through electronic meeting materials. Patients and primary caregivers are encouraged to discuss their own stressors and ways to relieve stress with researchers (*via* voice or text message or phone call).
Family Organization - Family treasure (Third week after surgery)	
Theme	Discover Available Resources
Form	Online family meeting
Target	Identify the family’s own strengths and available resources and learn how to use them. Summarize the experience of overcoming difficulties from past experiences.
Content	A brief recap of last week’s meeting. Combine pictures and words to show participants’ families what family resources, social resources and family strengths are. Help family members discover and demonstrate their family strengths and resources. Discuss success and failure cases and their characteristics in overcoming difficulties through family conversations, and summarize experiences. Discuss together how to leverage existing family strengths and resources to address current issues and challenges. Encourage different families to exchange experiences through WeChat groups and provide emotional support to each other.
Homework	Review the content of this meeting through electronic meeting materials. Give researchers examples of how their resources will be used in the future (*via* voice or text message or phone call).
Family Organization - Intimacy has a way (Fourth week after surgery)	
Theme	Improve Family Cohesion
Form	Online family meeting
Target	Create simple and effective family rules to promote family relationships and strengthen family bonds.
Content	A brief recap of last week’s meeting. Combine pictures, text and video to show patients and primary caregivers what family rules are and how they work, and encourage patients and families to share existing family rules. Through family discussions, according to the specific situation of each family, clear and effective family rules are jointly formulated to enhance family interaction, strengthen family bonds, and promote family relationships.
Homework	Review the content of this meeting through electronic meeting materials. Patients and primary caregivers share with researchers their family relationship status, understanding and feelings about family rules (*via* voice or text message or phone call).
Communication and Coping - Rational communication (Fifth week after surgery)	
Theme	Facilitate Effective Communication
Form	Online family meeting
Target	Understand what communication is and what communication problems families have. Learn effective communication skills.
Content	A brief recap of last week’s meeting. Combined with video, show a case of quarrel caused by improper communication. Combine pictures and videos to show patients and primary caregivers what communication is, and the types of family communication. Through family discussions, help participating families identify problems in family communication and why they arise. Introduce effective communication skills to participating families by combining pictures and videos.
Homework	Review the content of this meeting through electronic meeting materials. Patients and primary caregivers share a successful communication case with researchers (*via* voice or text message or phone call).
Communication and Coping - Through thick and thin (Sixth week after surgery)	
Theme	Family Problems and Coping
Form	Online family meeting
Target	Learn general ideas for problem solving. Develop a plan to address current family issues. Summarize the entire intervention process.
Content	A brief recap of last week’s meeting. Identify existing family issues through family discussions. Combine pictures, text, and video to show patients and their families the general steps to resolve family problems. Co-exploring solutions to existing family problems following the problem-solving approach. A brief summary of the entire intervention process.
Homework	Review the content of this meeting through electronic meeting materials. Patients and primary caregivers share a case of collaborative problem-solving with researchers (*via* voice or text message or phone calls).

### Instruments and measures

#### Basic information

Basic information of the study subjects, including sociodemographic information and clinical disease characteristics. Demographic information will include age, gender, education, religion, place of residence, marital status, monthly income, method of paying medical bills, occupation, and the patient’s relationship to the primary caregiver. Clinical disease characteristics will include disease stage, course, and primary treatment modality.

#### Outcome: Family resilience

The Chinese version of the Family Resilience Assessment (C-FRA) will be used to assess the family resilience level of the research subjects (Zhang et al., 2021). This scale was developed for family resilience of breast cancer patients and translated into Chinese. It has good reliability and validity in breast cancer patients. The scale contains 5 dimensions and 28 items measured on a 5-point Likert scale. The higher the score is, the better the level of family resilience. Cronbach’s alpha for this scale is 0.961.

#### Outcome: Coping style

The Coping Style Questionnaire (CSQ) will be used to assess the coping style of the research subjects (Xie, 1998). It has good reliability and validity in breast cancer patients (Xu and Fu, 2018). The questionnaire contains 2 dimensions and 20 items. The first 12 items are positive coping, and the last 8 items are negative coping. Each item is scored from 0 to 3. The total score is the standard score of the positive coping dimension minus the standard score of the negative coping dimension. A total score greater than 0 indicates that the individual has a mainly positive coping style, and a total score less than 0 indicates that the individual has a mostly negative coping style. Cronbach’s alpha for this questionnaire is 0.90.

#### Outcome: Social support

The Social Support Scale (SSS) will be used to assess the social support level of the research subjects (Liu et al., 2008). The scale has good reliability and validity in breast cancer patients (Liu, 2022). The scale contains 3 dimensions and 10 items. Items 1–4 and 8–10 each are scored from 1 to 4. Items 6 and 7 are multiple-choice questions, with 1 point for each option and 0 points for a mismatch. Items 5 A, B, C, and D options correspond to 1–4 points, respectively. A total score of 22 and below indicates a low level of social support, a score of 23–44 indicates a medium level of social support, and a score of 45–66 indicates a high level of social support. Cronbach’s alpha for this scale is 0.896.

#### Outcome: Family disease burden

The Family Burden Scale of Disease (FBSD) will be used to assess the family burden of disease in the families of the study subjects (Tsang et al., 2003). It has good reliability and validity in breast cancer patients (Feng and Wang, 2017). The scale contains 6 dimensions and 24 items. Each item is scored from 0 to 2. The standard score for each dimension is the total score for that dimension divided by the number of items in that dimension. A standard score greater than or equal to 1 indicates moderate or more family disease burden on this dimension. The higher the total score is, the heavier the family disease burden. Cronbach’s alpha for this scale is 0.956.

#### Outcome: Anxiety

The Self-Rating Anxiety Scale (SAS) will be used to assess the anxiety level of the research subjects (Tian et al., 2019). It has good reliability and validity in breast cancer patients (Zhou, 2014). The scale contains 20 items measured on a 4-point Likert scale. The cutoff value of the SAS standard score is 50 points: 50–59 points indicates mild anxiety, 60–69 moderate anxiety, and 70 points or more severe anxiety. Cronbach’s alpha for this scale is 0.777.

#### Outcome: Depression

The Self-Rating Depression Scale (SDS) will be used to assess the depression level of the research subjects (Tian et al., 2019). It has good reliability and validity in breast cancer patients (Zhou, 2014). The scale contains 20 items measured on a 4-point Likert scale. The cutoff value of the SDS standard score is 53 points: 53–62 points indicates mild depression, 63–72 moderate depression, and 73 points and more severe depression. Cronbach’s alpha for this scale is 0.782.

### Data collection

First, the researcher will introduce the purpose of the study to the patients and their families and establish a trust with them. After obtaining informed consent from patients and their families, their baseline data will be collected. Baseline data includes sociodemographic data, clinical disease characteristics, and psychosocial variables. Psychosocial variables include family resilience, coping styles, family disease burden, social support, levels of anxiety and depression. Investigators will measure psychosocial variables of participating families at four time points: baseline and 1 week, 1 month, and 3 months after intervention (Table 2). Participants will be assured that all data and intervention processes will be confidential. Only the main researchers will have access to the final trial dataset.

**Table 2:** Data collection note.

	Outcomes	Baseline	1 week post-intervention	1 month follow-up	3 month follow-up
Eligibility criteria	Demographic and clinical data	√			
Primary outcome	Family resilience	√	√	√	√
Secondary outcome	Coping style	√	√	√	√
	Social support	√	√	√	√
	Family disease burden	√	√	√	√
	Anxiety	√	√	√	√
	Depression	√	√	√	√

### Statistical analysis

Two investigators will enter and review data. SPSS23.0 will be used for data analysis with a one-tailed test and a significance level of 0.025. Descriptive analyses will be used for participants’ general data and the outcome variables, including means, standard deviations, and percentages. The t test or rank-sum test will be used for comparison if the baseline data of the two groups are continuous variables, and the chi-square test will be used for comparison if they are categorical variables. The effect of the intervention program will be assessed by observing changes in family resilience, coping styles, family disease burden, social support, anxiety and depression levels in the intervention and control groups. If the intervention effect within the group conformed to a normal distribution, the paired t test was used for evaluation; otherwise, the Wilcoxon rank-sum test was used. Repeated measures ANOVA will be used to assess the effect of the intervention at different time points between groups. AHP, multivariate adjustment analysis, and propensity score analysis will be used to control for confounding factors confounding the study results.

## Discussion

Research shows that family resilience interventions for families in adversity can help families better adapt and cope with challenges (Weine et al., 2003; Lim and Han, 2013; Inci and Temel, 2016; Miran, 2016). Therefore, appropriate interventions are needed to improve the resilience of families of breast cancer patients. At present, the research on family resilience intervention is still in its infancy and exploration stage, and there are differences and uncertainties in the effects of various family resilience interventions. The construction process of the intervention program lacks certain scientificity, and there is no report on intervention research on the resilience of families of breast cancer patients.

Therefore, we focused on the coping process of Wu Dy′s resilience process model of families of breast cancer patients and take Walsh’s family resilience theory as the framework to form the first draft of the family resilience intervention plan for breast cancer patients through literature analysis (Walsh, 2003; Wu, 2020). Then, the form and content of the intervention plan were adjusted through structured interviews with patients, family members, and medical staff in breast cancer-related departments. In addition, experts in related fields were invited to adjust the content of the intervention plan several times to form the final draft of the intervention plan. Compared with some intervention studies on family resilience, the construction process of this intervention program is more scientific and reasonable.

In addition, this intervention program is focused on the coping process, with belief systems, organizational patterns, and communication/problem-solving processes as the framework, and it comprehensively intervenes in the families of Chinese breast cancer patients by addressing six aspects: disease, attitude, resources, rules, communication and problem solving. This intervention is more comprehensive than other family resilience interventions that target only one aspect of family resilience, such as family communication or family problem solving. If this intervention proves to be effective, a comprehensive intervention for resilience in families of breast cancer patients will be recommended. Help these families make comprehensive and effective adjustments in awareness, behavior, and family cooperation, actively adapt to and deal with various problems caused by the disease and provide a practical basis for the clinical nursing of breast cancer patients and their families.

Finally, this research protocol proposes a one-on-one online family meeting approach to provide resilience interventions to each family in the participating group. Compared with the multifamily group meeting adopted by other family resilience interventions, it is more suitable for the Chinese cultural context. In addition, online meetings can reduce the burden on families of breast cancer patients, prevent the risk of COVID-19 infection and is more suitable for the overall care needs of patients and their families. If the intervention proves to be effective, this form of intervention could also be applied to the home care of patients with other medical conditions.

### Limitations

This study may have some limitations. First, there are many family meetings in the intervention program, and subjects may not be able to attend all of them due to physical or other reasons, which will lead to sample loss that may have an impact on the study results. Second, because of the nature of the study, it was not possible to blind the interventionists. Therefore, this study will arrange for independent researchers to collect and evaluate the data. Finally, while each family meeting will be held in an isolated quiet setting, some subjects may be contaminated due to subject recruitment and the first family meeting being held in the same department of the hospital. Therefore, when arranging meetings for families in the intervention group, researchers should try to choose an appropriate place and time to avoid crowd gathering and contamination between study subjects.

## Conclusion

This study aimed to demonstrate a coping-focused family resilience intervention to increase the level of family resilience in families with breast cancer and help them cope better with adversity. If proven effective, the intervention will provide a new rationale for clinical research, as it is the first coping-focused family resilience intervention for breast cancer patients. It will also help guide breast cancer patients and their families to successfully manage the disease. If the program is not effective, professional support can also be provided to the families of the intervention group to help them better cope with the disease. At the same time, it can accumulate experience for the further improvement of the later intervention program and provide a empirical basis for future implementation.

## Data availability statement

The original contributions presented in the study are included in the article/supplementary material, further inquiries can be directed to the corresponding author.

## Ethics statement

This study was approved by the Ethics Committee of Anhui Medical University (Ethics Number: 20190267).

## Author contributions

JG and J-XL participated in the research conception and design. Data collection and analysis was performed by J-YS and W-YC. H-PL, M-KZ, and S-SZ drafted and revised the manuscript. All authors participated in data interpretation, draft review and approval of the final manuscript. Everyone is ultimately responsible for the quality of the content and the decision to submit for publication.

## Funding

This research is supported by the Scientific Research Foundation of Education Department of Anhui Province of China (No. KJ2020A0221) and Anhui Medical University School of Nursing Seedling Cultivation Project (hlqm2021044). Funders are not involved in the development of this research.

## Conflict of interest

The authors declare that the research was conducted in the absence of any commercial or financial relationships that could be construed as a potential conflict of interest.

## Publisher’s note

All claims expressed in this article are solely those of the authors and do not necessarily represent those of their affiliated organizations, or those of the publisher, the editors and the reviewers. Any product that may be evaluated in this article, or claim that may be made by its manufacturer, is not guaranteed or endorsed by the publisher.
