# Corrigendum: A randomized controlled trial of a coping-focused family resilience intervention program for breast cancer patients: study protocol

**DOI:** 10.3389/fpsyg.2023.1240505

**Published:** 2023-06-26

**Authors:** Jie Gao, Jia-Xin Li, Wei-Ying Chen, Jiang-Yan Song, Meng-Ke Zhou, Shan-Shan Zhang, Hui-Ping Li

**Affiliations:** ^1^School of Nursing, Anhui Medical University, Hefei, Anhui, China; ^2^Department of Nursing, Anhui Provincial Hospital, Hefei, Anhui, China; ^3^Department of Nursing, Clinical College of Anhui Medical University, Hefei, Anhui, China

**Keywords:** breast cancer, family resilience, intervention, protocol, randomized control trail

In the published article, there was an error regarding the affiliation(s) for Wei-Ying Chen. As well as having affiliation 2, they should also have affiliation “^1^School of Nursing, Anhui Medical University, Hefei, Anhui, China”.

The authors apologize for this error and state that this does not change the scientific conclusions of the article in any way. The original article has been updated.

